# Some Remarks on the Pathogenesis of Tuberculosis*Read before the Fifth District Medical Association, April 1, 1909, at San Antonio, Texas.

**Published:** 1909-09

**Authors:** Theo. Y. Hull

**Affiliations:** San Antonio, Texas


THE
### TEXAS MEDICAL JOURNAL.
Established July, 1885.

F. E. DANIEL, M. D., - - - - Editor, Publisher and Proprietor

PUBLISHED MONTHLY.—SUBSCRIPTION $1.00 A YEAR.

VOL. XXV. AUSTIN, SEPTEMBER, 1909. No. 3.

The publisher is not responsible for the views of contributors.

### Original Articles.

For Texas Medical Journal.

### Some Remarks on the Pathogenesis of Tuberculosis.*

BY THEO. Y. HULL, B. S., M. D., SAN ANTONIO, TEXAS.

In discussing any phase of tuberculosis before a body of physicians, I am aware that I am open to the criticism of telling "twice told tales"; but those of us engaged in this anti-tuberculosis work, which might appropriately be called "rescue work," are so enthusiastic that any new idea or modification of an old idea is of perennial interest. In tuberculosis we are dealing with a disease so old that it antedates history, so universal that no land, no clime, no people is exempt, and one in which the traditional "ounce of prevention" has more than its usual significance. Preventive medicine has in the tuberculosis field its greatest opportunity for good, for, while yellow fever and variola, thanks to its efficacy, are practically of little significance, the "white plague" still reaps an undiminished harvest in human lives. But in applying successfully preventative measures to this disease, it is most important that we should have an intelligent conception of its pathogenesis. I believe that much of our effort to stamp out tuberculosis, while made in the deepest sincerity, has been largely nullified by the limitation of our knowledge.

*Read before the Fifth District Medical Association, April 1, 1909, at San Antonio, Texas.

### CERTAIN PRINCIPLES IN INFECTION.

In this paper I wish to direct your attention to certain phases in the tuberculous process that should give us a clearer conception of the struggle waged by the inherent powers of the human body against its parasitic enemy, the tubercle bacillus, and direct us in establishing a rational prophylaxis.

(1) We know that the disease is due to the growth of the tubercle bacillus within the living tissues. We should understand that the mere presence of the organisms does not constitute tuberculosis, but, under certain physical conditions, they may set up the tuberculous process. When we consider the large proportion of contaminated food—meat and dairy products—consumed, there is a high probability that were sufficient search made, virulent bacilli would be found in the blood of a large per cent of the human family, when, as a matter of fact, only a relatively small per cent die therefrom. Infection requires, therefore, a sufficient quantity of virulent bacilli and a depressed condition of the tissues.

(2) The normal tissues, and especially the blood, are inimical to the growth of the tubercle bacillus. That the blood possesses marked germicidal powers to certain organisms is well known. In typhoid fever and in cholera there is decided bacteriolysis. This is true in tuberculosis, but to what extent we do not know. It is highly probable that a few isolated bacilli would be quickly disposed of by the normal tissues. This power is apt to be in direct proportion to the immunity established. Every one familiar with the treatment of tuberculosis has observed that as cases progress toward recovery, the number of bacilli in the sputum decrease, and in the event of recovery, disappear altogether. With the improved methods of Rosenberg, we will, in the future, be able to determine whether they are eliminated from the blood. When clinical recovery occurs, the patient assumes the appearance of health, the agglutinating power of the blood serum increases, likewise its alkalinity. In this case it has recovered its normal properties and inhibits the activity of the micro-organisms, if it does not directly destroy them as in typhoid fever and cholera. The following case seems to indicate direct bacteriolysis of the tubercle bacillus in recovery from tuberculosis: Mr. G. was treated several years ago for active pulmonary tuberculosis and apparently made a complete recovery. He returned five years later. His symptoms pointed to a renewal of the tuberculous process. The microscopic examination of the sputum revealed the presence of a few pneumococci, many influenza bacilli, streptococci and staphylococci; but no tubercle bacilla were found at that time nor at any time during the next two months, when he died from pulmonary abscess and empyema. The microscopic examination of the pulmonary tissues failed to discover the presence of the tubercle bacillus.

(3) The tubercle bacillus grows with difficulty except under favorable conditions. The healthful body does not afford such favorable conditions. It is highly probable that if the blood be maintained in a normal state it will destroy them or inhibit their activity unless they be present in great abundance or of unusual virulence. Post-mortem examinations show that a very large per cent of all persons over 18 years of age, dead from other causes, have healed tuberculous lesions. This can not be explained unless we assume that the normal blood has some bacteriolytic property.

(4) We thus see that for infection to occur, the natural power of defense possessed by the body, either local or general, must be weakened. With lowered resistance, the tubercle bacillus may be deposited by the inspired air, the lymphatics or the blood current in some spot where the vitality of the cells has been impaired and the tuberculous process results. That the lowered vitality invites infection by creating a favorable soil is seen in the frequency with which tuberculosis follows typhoid fever, influenza and other acute infectious diseases.

### SIGNIFICANCE OF PULMONARY HEMORRHAGE.

In this connection, I wish to refer to certain phenomena in relation to pulmonary hemorrhage. Every physician with considerable experience in the treatment of tuberculosis had undoubtedly noticed a considerable improvement, and sometimes a permanent improvement, follow such an event. You will even hear the laity speak of pulmonary hemorrhage as a favorable sign. I have recently observed sudden improvement follow an intestinal hemorrhage in typhoid fever. This observation, together with the discovery that the blood possesses decided germicidal power, which, outside of the vessels seems greater than within, led to the belief that many bacilli were destroyed during the hemorrhagic process. This destruction of bacilli within the tuberculous foci is probably true only to a limited extent, and it could have little or no effect upon the patient if it were true. A more probable reason for improvement lies in the fact that tuberculosis is both a toxemia and a bacteremia. During a moderate hemorrhage a large number of bacilli and their toxins are swept out of the body. The blood, thus relieved of a certain per cent of its load of bacteria and toxic substances, is able to recover its loss of blood corpuscles much more rapidly than the bacilli can multiply and elaborate their toxins. This increase in the relative proportion of blood corpuscles to micro-organisms increases its bactericidal power, thus rendering the conditions less favorable to the invading bacteria, and more favorable for recovery. In this light the old practice of "blood-letting" had some basis in truth.

### CHANNELS OF INFECTION.

Nearly all text-books devote long articles to heredity and hereditary predisposition. After all, what does heredity signify? It means that parents weakened by chronic disease can not beget healthy children, and the great masses of data to prove this and that contention, simply indicate that for the children of tuberculous parents, the chances of infection are greatly multiplied. Above I referred to the presence of tubercle bacilli in the blood of infected individuals. To explain prenatal infection without placental disease it has long been urged that the bacilli must circulate in the blood. In this connection, heredity has considerable significance, but as a channel of infection it is of little importance. We may, therefore, direct our attention to the mucous membrane of the respiratory and digestive systems. The infective agent must be contained in the air or food. For years the theory of infection by inhalation has had few opponents, and practically all of our legislation has been directed from this viewpoint. But of late a school of physicians has arisen who insist that we should not ascribe so large a per cent of the cases of infection to the inhalation of virulent organisms. Some even hold that it is of minor importance.

However this may be, the discussion has led to renewed investigations and some new ideas are developing. At first thought we are almost certain to adopt the air theory of infection because it is quite apparent to our senses that it should be so. When we see the number of cases arising among factory employes, office clerks and others whose occupation compels them to inhale to a greater or less extent a dust-laden atmosphere, when we realize that the dust of a business street may contain as high as six million bacteria per cubic centimeter, all of which are not harmless—and further, when we know that a single tuberculous individual may in twentyfour hours throw off from one to five billion bacilli, we are prone to believe the highest percentage not exaggerated. On the other hand, a study of the natural defense of the respiratory tract—the peculiar structure of the bronchial epithelium and its perfect adaptation to the purpose; and the further fact that relatively few micro-organisms of any kind are ever found even within the smaller bronchioles—is certain to cause one to reflect upon the possibility of error.

The conversion of the ciliated columnar cells of the terminal bronchioles into the cuboidal cells of the alveolar ducts, the flat polygonal cells of the infundibula—the respiratory epithelium—and the endotheloid plates and stomata of the alveoli indicates that nature did not contemplate the invasion of the deeper recesses of the lungs by bacteria—the only portion where infection can be easily accomplished. In this light the contention of Calmette and others that "neither dust nor microscopical particles of sputum can find their way into the deeper portions of the lungs with the inhaled air," does not seem entirely unreasonable. On the other hand, it may be argued that nature provided the leucocyte—a potent agent for either defense or infection—and the stomata for the very purpose of freeing the alveoli from irritating particles inhaled. Anthracosis may be cited as further evidence that particles of dust do reach the alveoli. Of the numerous experiments undertaken to prove the possibility of infection by certain channels, all have been open to the criticism of being more or less imperfectly carried out, thus destroying their value as scientific evidence. Probably the most successful experiments have been those of Svensson, a Swedish physician, who, in attempting to prove the impossibility of infection by inhalation, not only proved its possibility, but its certainty. In this, the scientific proof, not only that the bacilli enter the lungs, but all parts of the lungs, was found.

The fact that many tubercles are found just within the alveoli at a point where the dust would be most likely to lodge, is cited as an evidence of infection by inhalation; but Osler claims that this is proof of infection through the circulation, and that the site of infection by inhalation is in the bronchioles. The advocates of this mode of infection admit that, under normal conditions, few or any bacteria can reach the infundibular region; but, with the prevalence of inflammatory diseases of the respiratory tract, and the forced breathing consequent upon coughing, particles of mucus are frequently carried into the alveoli, where the organisms are attacked by the leucocytes, engulfed and carried within the lymph channels—to be destroyed, or in turn destroy the leucocytes. Its lodgment at this point within the mouth of the alveoli would give rise to the primitive tubercle where it is most frequently found. This argument would seem unsurmountable if the same thing could not be proven to occur in an entirely different manner. In cattle, the infection is admittedly by inhalation. Nearly 100 per cent of all cattle infected have pulmonary tuberculosis. Only 70 per cent of the swine infected have pulmonary tuberculosis. The route of infection here is admittedly by ingestion. Those who have given much study to the prevalence of tuberculosis among domestic animals must not be condemned for thinking very seriously of the gastro-intestinal route of infection. Not only is contaminated food swallowed, but a very large per cent of the inhaled dust finds its way into the stomach. It is contended that the organisms would be destroyed by the gastric juice, or would be unable to pass through the normal mucous membrane. Actual experiments show both contentions wrong. The tubercle bacillus will resist the action of the gastric fluids from eighteen to thirty-six hours, and they have been found within the contents of the thoracic duct a few hours after ingestion. The ingestion theory is supported by the experiments of Calmette, Guerin, Vallee, Barthel and others who hold that infection almost always occurs as a result of the infective agent reaching the alimentary canal, passing through the mucous membrane, the lymphatic glands and ducts, and carried by the blood current directly into the lungs. Behring believes that tuberculosis originates in this way in practically all cases. He affirms—and many students agree with him—that infection occurs during the early years of life from contaminated food—principally milk—and remains latent for years, even decades.

Many writers hold that tuberculosis is primarily a lymphatic disease, and that the infective agent, derived from whatever channel, finds its first resting place within the lymph glands. Barthel in particular urges the existence of a "lymphoid stage." He believes that infection from the pharynx, stomach and intestines is far more frequent than has been generally considered. The frequent occurrence of infected cervical glands in children, the direct connection of these glands with the lymph ducts and blood current, and the experimental evidence that the tubercle bacillus can readily pass through the normal pharyngeal membrane, should direct our attention to the mucous membrane of the upper air passages as a portal of entrance to pathogenic bacteria. In this connection the importance of diseased tonsils and pharynx should not be disregarded, for, whether the infective agent be in the dust or the food, infection is liable to occur, and a diseased membrane invites infection.

In our study of infection, certain things become evident, that may aid us in determining the relative importance of the air tubes, the lymphatics and the blood vessels as routes of invasion. Statistics reveal that 90 per cent of the cases of tuberculosis are pulmonary. Some urge a special susceptibility to infection. A careful study of facts must convince us that the lungs are no more predisposed to tuberculosis than any other part of the human body. We must conclude, however, that the opportunity for pulmonary infection is vastly greater. Animal experimentation is of great value in determining the greater frequency of pulmonary lesions. The experiments upon rabbits, already referred to, show conclusively that infection can take place by inhalation. Experiments upon dogs and other animals demonstrate the passage of tubercle bacilli through the normal mucous membrane of the digestive tract into the lymph channels to be taken up and deposited of necessity in the lungs. The part played by the lymphatics in pulmonary infection is clearly shown in the following experiments. The rubbing of tubercular matter into the skin of the nose and cheek caused lesions in the following order: (1) Cervical glands; (2) bronchial glands, and (3) lungs. Infected material rubbed into the nasal mucous membrane produced precisely the same results in the same order. The rubbing of cultures of tubercle bacilli upon the membrane of the mouth, tongue, tonsils and pharynx gave such uniform results as to leave no possible doubt of the role of the lymphatics in pulmonary infection. Subcutaneous injection of bacilli in the hypogastric region gave the following involvement: 20 days, inguinal glands; 30 to 40 days, spleen; 40 days, liver; 40 to 50 days, bronchial glands and lungs. We thus see that the specific agent taken up by whatever channel, invariably reaches the pulmonary tissue, which does not require any special susceptibility to become the seat of disease.

### WHY APICAL INFECTION?

In this connection another question arises: Why are the apices so frequently the seat of the primary involvement? This has been said to be due (1) to especial apical susceptibility; (2) to deficient expiratory activity in the apices, and (3) to an enfeebled circulalation and poor nutrition. The fact of apical infection must be capable of a purely physical explanation. There is not a scintilla of evidence to prove that any part of the lung is any more susceptible to infection than any other part of the body. The lung is an elastic organ. The air pressure is the same in all parts, and, therefore, the relative expansion is the same everywhere, and consequently the circulation and nutrition is equally good in all parts. Calling the apex a "locus minoris resistentiae" does not explain it; for, why should the apex be less resistant to the tubercle bacillus and more resistant to the pneumococcus than other portions of the lungs? Again, if the circulation is less active in the apex than elsewhere, why do we find recovery much more frequent from infection of the upper lobes than the lower? Nutrition must have much to do with recovery. The exponents of the inhalation theory claim that a larger portion of dust collects in the upper lobes. As a matter of fact, a larger per cent of the inhaled dust never reaches the lungs at all, and the major part of what does is collected in the lower lobes. Why should the pneumonia germ fall in one part and the tuberculosis germ in another part with equal certainty? To the casual observer the law of gravitation should work equally well in all cases. It is clearly evident, therefore, that a more rational explanation is demanded. We are thus driven to the conclusion that the blood vessels and lymphatics convey the infective agent in a large per cent of all cases of pulmonary tuberculosis. This, the "embolic theory" of infection, we believe to be based upon certain anatomical features of the heart and the pulmonary artery and its branches, together with certain physical laws. The course of the pulmonary artery and its branches are worthy of study. This vessel, about one and one-half inches in length, arises from the right ventricle, passes upward, backward, and slightly to the left, passing behind and beneath the aorta, where it subdivides into the right and left pulmonary arteries. The right pulmonary artery, slightly larger than the left, passes outward and subdivides into a large upper branch, which supplies the upper and middle lobes, and a smaller branch, which distributes blood in the lower lobe. It will be remembered that the right lung is somewhat larger than the left, and, therefore, aerates a somewhat larger portion of the blood. The left pulmonary artery passes outward to the left and subdivides into an upper and a lower branch which distribute the blood to the upper and lower lobes, respectively.

It will be noticed that the direction of the blood current on leaving the right ventricle, together with the larger caliber of the right pulmonary artery, conspire to throw a stronger and larger current of blood into the right lung. Again, the upper branch of the right pulmonary artery, being larger than the lower, permits a correspondingly larger portion of the blood to be sent into the right lobe. The left pulmonary artery pursues a somewhat similar course with the exception that the upper branch is not perceptibly larger than the lower.

The ventricular contraction throws the blood into the main artery with considerable force, which, according to the laws of physics, will pursue the path of least resistance. The right artery being slightly larger and more in line with the current on leaving the ventricle, will receive more than its fellow. Again, the upper branch will, on account of size, receive a more abundant supply.

Now, if the embolic theory of infection be true, a mass of tubercle bacilli or even particles of carbon or what not—derived from the intestinal tract, the cervical or mesenteric glands or some caseating lymph-nodes be thrown into the blood stream, it will of necessity reach the pulmonary rather than the systemic circulation. The embolus being projected from the right ventricle, the chances are that its own momentum, the direction of the force exerted, and the stronger current will conspire to carry it more frequently into the right than to the left lung. This will agree with our observation of greater frequency of infection in the right lung, simply because of greater opportunity.

Again, the embolus being heavier than the blood, would require greater momentum, and be, therefore, less easily deflected from a straight line, and would of necessity impinge upon the upper internal surface of the artery and escape into the branch supplying the upper or apical lobe on both sides, rather than into the lower branches. The infective material, as I have shown before, is arrested at the point where the relatively large branches break up into the fine capillary net work of the alveoli and explaining the occurrence of the primitive tubercle just within the mouth of the alveoli.

As thus set forth, and as claimed by its advocates, the embolic theory of infection gives a rational explanation of the greater frequency of pulmonary infection over other organs of the body, the greater frequency of the lesion in the right lung than the left, the selection of the apical rather than the lower lobes, and the site of the primary tubercle at the mouth of the alveoli.

In conclusion, I wish to state that I am not here to argue for or against either theory of infection. The evidence produced by animal experimentation proves conclusively that the infective agent—the tubercle bacillus—may be and is taken into the system with the air we breathe and the food we eat. My own observations, however, lead me to believe that infection takes place far more frequently through infective material swallowed—such as meat, milk and butter—than we have been hitherto willing to admit. In fact, it is becoming more and more evidence that we should give more attention to our food supply.

We are now attacking the tuberculosis problem from all sides. We must fight not only to maintain the ground already won, but to advance. It is time we understood the relative importance of the various channels and agencies of infection. More study should be devoted to them. The importance of correct conclusions should not be underestimated. When such men as Behring, Calmette and others of national and international reputation advocate certain principles, they do so only after due study and deliberation. The views they hold are of vast commercial significance. If infected food is causing any great number—in fact any number—of cases of tuberculosis—and very many of us believe a large per cent due to this cause—it is time we know, and set about securing proper legislation. There can be no letting up in the fields we are now working, but we must push our campaign vigorously, and into some new fields. Registration, disinfection, sanitation and general enlightenment we must have; clean homes and clean patients we should have, and it is equally important, nay, imperative, that we should know beyond peradventure, that the food we partake of and our loved ones partake of is free from the infective agent of the most universal and most dreaded of all maladies, tuberculosis.

